# Effectiveness of 13-valent pneumococcal conjugate vaccine on all-cause pneumonia in children under 5 years in Shanghai, China: An observational study

**DOI:** 10.1016/j.vaccine.2023.08.041

**Published:** 2023-09-22

**Authors:** Jie Tian, Bo Zheng, Laibao Yang, Ying Guan, Chunze Xu, Weibing Wang

**Affiliations:** a School of Public Health, Shanghai Institute of Infectious Disease and Biosecurity, Fudan University, Shanghai 200032, China; b Department of Immunization, Pudong New Area Center for Disease Control and Prevention, Shanghai 200136, China; c Department of Health Information, Songjiang Center for Disease Control and Prevention, Shanghai 201620, China; d Key Laboratory of Public Health Safety of Ministry of Education, Fudan University, 138 Yi Xue Yuan Road, Shanghai 200032, China

**Keywords:** *Streptococcus pneumoniae*, PCV13, Cox proportional hazards regression, All-cause pneumonia

## Abstract

**Background:**

*Streptococcus pneumoniae* (*Spn*) is a common respiratory pathogen and the main cause of bacterial pneumonia, meningitis, and bacteremia, acute otitis media. Imported 13-valent pneumococcal conjugate vaccine (PCV13) was licensed in China and introduced in Shanghai in 2017. We aim to describe PCV13 vaccination trends and pneumonia incidence of children under 5 from 2017 to 2020, then estimate the effectiveness of PCV13 against community-acquired pneumonia (CAP) in children under 5 in Shanghai, China.

**Methods:**

By calculating propensity scores with logistic regression, a comparison group was formed by frequency matching one unvaccinated child to one vaccinated child. For matching, we used the nearest-neighbor matching algorithm and exact matching, and then created distinct matched analysis sets for two cohorts. A Kaplan-Meier analysis was conducted to measure the cumulative incidence of all-cause pneumonia in both groups and used the log-rank test to assess the differences between the two cumulative incidence curves. Cox proportional hazards regression was used to compare the adjusted hazard ratios (HR) of differences in all-cause pneumonia between the two groups.

**Results:**

Children received three or more doses PCV13 accounted for 85.7% of all vaccinated children. The incidence of pneumonia in Shanghai’s Songjiang district decreased rapidly from 2017, when PCV13 vaccination presented an overall increasing trend. The estimated vaccine effectiveness against visits for all-cause pneumonia was 19% (95% CI: 3 to 32) after the first dose in children vaccinated with at least one dose of PCV13. The protective effectiveness of PCV13 was found to be higher for hospitalized pneumonia (30%, 95% CI: 5% to 49%) than for outpatient pneumonia (19%, 95% CI: 4% to 32%).

**Conclusions:**

PCV13 vaccination among children aged 0–5 years substantially reduced the incidence of all-cause pneumonia. Direct immunization of children under 5 years is an effective strategy to combat outpatient pneumonia, and hospitalized pneumonia.

## 1 Background

**Fig. 1:**
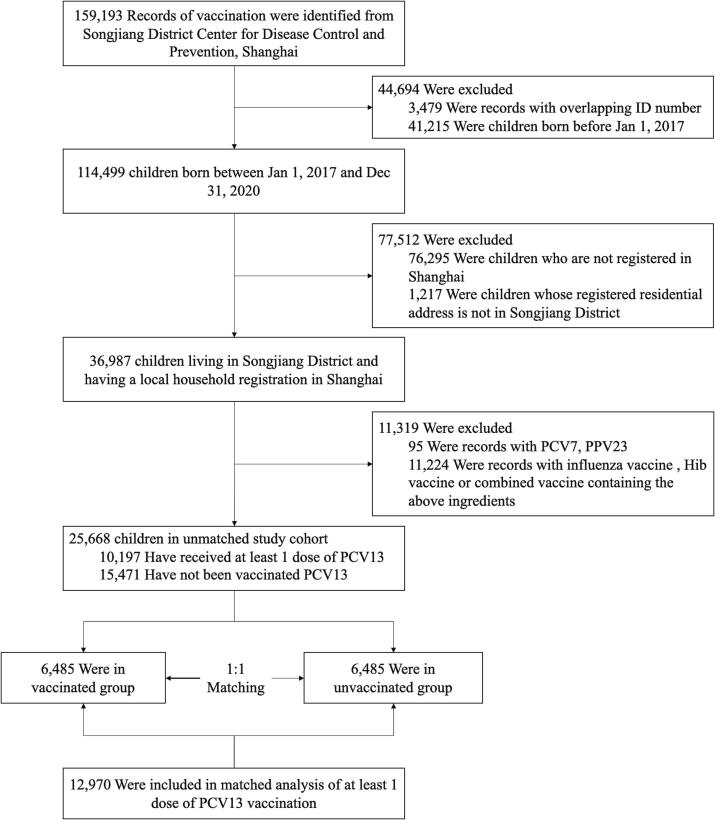
Construction of cohorts with and without PCV13 vaccination among 0- to 5-year-old children matched by propensity score. A total of 159,193 vaccination records as of December 31, 2020 were obtained. Records of non-Shanghai Songjiang District household registration, and records of vaccinated influenza vaccine, PCV7, PPSV23 and combined vaccines containing the above vaccine components were excluded. Then those records were grouped and matched according to whether they had been inoculated with PCV13.

*Streptococcus pneumoniae* (*Spn*) is a Fig. 1 common respiratory pathogen and the main cause of bacterial pneumonia, meningitis, and bacteremia, acute otitis media [1]. It is ranked by the World Health Organization (WHO) as one of 12 key pathogens that cause a heavy burden of disease [2], [3]. Pneumococcal disease, which is among the most severe public health issues worldwide, remains a significant cause of infection associated with high mortality and morbidity in children and adults [4], [5]. China has a high burden of pneumococcal diseases, with approximately 12% of total cases worldwide occurring in the country and more than 30,000 children 1–59 months of age dying each year from invasive pneumococcal disease [6]. In particular, community-acquired pneumonia, which is the leading clinical disease caused by *Streptococcus pneumoniae* infection [7], causes substantial morbidity and mortality worldwide and generates considerable healthcare costs [8]. Between 1980 and 2008, there were 12,815 cases per 100,000 individuals per year of all-cause pneumonia among children aged between 1 and 59 months, with 526 deaths per 100,000 such children recorded annually in China [9].

Pneumococcal conjugate vaccine (PCV) is one of the most effective measures to prevent pneumonia [10]. It has been developed and implemented to reduce the burden of disease among children. The first pneumococcal conjugate vaccine, Prevenar (PCV7), acted against serotypes 4, 6B, 9V, 14, 18C, 19F and 23F and became available in 2001 [11]. It was introduced in China in 2008, only available of the private market, but was pulled from the market in 2015 due to the expiration of its import license [12]. The new 13-valent PCV (PCV13) includes the serotypes targeted by PCV7 plus six more pneumococcal serotypes; these 13 serotypes include the most prevalent serotypes among invasive cases of disease in China [13]. The WHO recommends that all countries adopt programs to provide universal pneumococcal vaccination to infants, the elderly, and other vulnerable populations [14]. Meanwhile, The Global Alliance for Vaccines and Immunization (GAVI) has provided funding for pneumococcal vaccines in low-income countries throughout Africa and Asia [15]. Despite the high vaccine cost, some middle-income countries (e.g., South Africa and Brazil) have added a pneumococcal vaccine to their national immunization schedule [16].

Currently, pneumococcal vaccines are not classified as a mandatory vaccination for infants under the Chinese government’s Expanded Program for Immunization (EPI). There is potential to reduce the high burden of pneumococcal diseases (e.g., CAP) in China by introducing a compulsory infant pneumococcal vaccination program through the EPI. Globally, many countries introduced PCV have conducted observational studies on the effect of PCV [17], [18], [19], [20]. Since PCV was launched in China, there have been no large-scale real-world studies about its effectiveness. Several large-scale clinical trials have demonstrated the clinical efficacy and safety profile of PCV in infants and children [21], [22], [23]. However, there has been some debate as to whether this type of program would be cost-effective, and the results of the relevant studies have been mixed in light of the WHO’s recommended thresholds for cost-effectiveness [24], [25], [26], [27]. Moreover, most of the existing studies were based on PCV7, which is no longer manufactured and is being replaced by newer vaccines [28]. The introduction of a PCV into the National Immunization Program (NIP) needs to be supported by a stronger evidence base, including data on pneumococcal disease burden (pneumonia, meningitis, and otitis media) and the economic burdens related to these diseases. Therefore, post-marketing evaluation of vaccine effectiveness in China is urgently needed.

Imported PCV13 was licensed in China and introduced in Shanghai in 2017. The cost of adding PCV13 to the infant vaccination schedule should be considered alongside its potential to prevent pneumococcal disease and related cost burdens. As limited data are available on the effectiveness of PCV13 in China, we herein sought to estimate the effectiveness of PCV13 against CAP in Shanghai, China, and provide data that may be used in the future for cost-effectiveness analysis of PCV13.

## 2 Methods

### 2.1 Study aim and data source

This study is a retrospective cohort study aimed to describe PCV13 vaccination trends and pneumonia incidence of children under 5 from 2017 to 2020 and evaluate the effectiveness of PCV13 on all-cause pneumonia in children under 5 years of age in Songjiang district of Shanghai, China. Songjiang district is located in the suburbs of Shanghai and have a very complete and efficient medical information management system. The demographic data (including the number of births per year, the registered population of different age groups) for Songjiang District in Shanghai were derived from the Shanghai Statistical Yearbook. We obtained the vaccination records and medical records of children born between January 1, 2017 and December 31, 2020 through the Songjiang District Center for Disease Control and Prevention, Shanghai.

### 2.2 Study design and participants

Using vaccination data related to the immunization program, we identified all children born in Shanghai’s Songjiang District between January 1, 2017 and December 31, 2020. Based on whether the PCV13 vaccine has been administered (including domestic or imported vaccines), all vaccination records were divided into a vaccination group and a non-vaccination group. Children who were vaccinated with any dose of PCV13 in our study were placed in the vaccination group, which was further subdivided into a complete vaccination group (3 doses of PCV13 plus one booster dose of PCV13 [3p + 1b], 2p + 1b, or 3p) and an incomplete vaccination group (1p + 1b, 1p, 2p, or 1b) according to the vaccination procedure.

The outcome was clinical pneumonia based on the ICD-10 codes, which were assessed and determined by specialist physicians according to standard clinical criteria. Since not every patient with pneumonia undergoes etiological examination, although the ICD10 code specifically sets the classification of bacterial pneumonia and pneumococcal pneumonia, doctors may just generally classify it into pneumonia classification. To avoid possible attrition bias, we included all pneumonia-related codes (ICD-10J12-J18) as this study’s outcomes. The time of the first clinical pneumonia diagnosis between 2017 and 2020 served as the diagnosis time. The follow-up person-years were calculated for each child until they were diagnosed with all-cause pneumonia, withdrawn from the system, or censored because of death. Children with a history of all-cause pneumonia before vaccination or for whom information was incomplete were excluded. Inpatient pneumonia refers to patients who need to be hospitalized for treatment, but not hospital-acquired pneumonia.

### 2.3 Exclusion criteria

We excluded records missing the date of birth, sex, household registration type, and/or residential address. We also excluded records that were suspected of having registration errors (for example, a child listed as receiving two second doses) after the data were logic-checked. Since other types of pneumonia vaccines and influenza vaccines can also have a protective effect on the occurrence of pneumonia in children under 5, to avoid possible cross-protection effects, we also excluded records of those who were vaccinated with 23-valent pneumonia vaccine, influenza vaccine, *Haemophilus influenzae* type b vaccine, and other vaccines containing the above three vaccine components. During the study period, PCV13 was the sole PCV vaccine recommended by the Chinese national vaccination program. Few children who were previously vaccinated with PCV7 were excluded from our study.

### 2.4 Statistical analysis

To control for potential confounders, we calculated propensity scores using logistic regression [29], [30], [31], which estimated the probability of being vaccinated with PCV13 for all children, given all baseline characteristics. A comparison group was formed by exactly matching one unvaccinated child to one vaccinated child according to age, sex, type of household registration (local or foreign), residential street, and propensity score. This generated a distinct matched analysis set for the two cohorts. To assess the balance of covariates achieved by this matching, we evaluated standardized differences between the vaccinated and unvaccinated groups. We considered covariates with a standardized difference of<10% to be well balanced.

The date of the first dose PCV13 vaccination was designated as the underlying time scale to enable us to measure follow-up person-years. Hazard ratios with 95% confidence intervals (CI) were estimated with the use of Cox proportional-hazards regression. The Wald test was used to assess goodness of fit of the Cox proportional hazards model, which was achieved for the analyses of outpatient pneumonia and inpatient pneumonia.

We conducted a Kaplan-Meier analysis to measure the cumulative incidence of all-cause pneumonia in both study groups, and used the log-rank test to assess the differences between the two cumulative incidence curves. Cox proportional hazards regression was used to compare the adjusted hazard ratios (HR) of differences in all-cause pneumonia between the two groups at a 95% CI. Sex, age, and household registration were controlled for proportional risk assumption. We calculated the 95% CI using the percentile bootstrap method with 1,000 repetitions.

The R software (R Foundation for Statistical Computing, Vienna, Austria, version 4.0.5) was used to perform statistical analyses, and *P* <.05 in two-tailed tests was taken as indicating statistical significance.

## 3 Results

### 3.1 Cohort characteristics

During the study period, we identified 159,193 records of vaccination, of which 25,668 were eligible for inclusion in the study cohort. Before matching was performed, children in the cohort were unevenly distributed by gender, age, and place of residence. In the unmatched cohort, the age distribution was statistically different, with younger children being more vaccinated. In gender distribution, females were slightly more vaccinated, but the difference was not statistically significant. After we performed individual matching with a 1:1 ratio, the demographic characteristics were well balanced between the vaccinated and unvaccinated groups (Table 1).

**Table 1:** Characteristics of unmatched vaccinated newborns in Songjiang District, Shanghai, 2017–2020.

Characteristics	level	Unmatched cohort				Matched cohort of at least 1 dose PCV13 vaccination			
		Overall (N = 25,668)	Unvaccinated Controls (N = 15,471)	Vaccinated Children (N = 10,197)	*p*	Overall (N = 12,970)	No Vaccine Exposure (N = 6,485)	Vaccine Exposure (N = 6,485)	*p*
Age (mean (SD))		25.44 (13.29)	27.62 (13.82)	22.12 (11.68)	<0.001*	24.62 (11.99)	24.63 (11.99)	24.60 (11.98)	0.906
Age group (%)	0 to 11 m	4767 (18.6)	2513 (16.2)	2254 (22.1)	<0.001*	2192 (16.9)	1096 (16.9)	1096 (16.9)	0.998
	12 to 23 m	6715 (26.2)	3355 (21.7)	3360 (33.0)		3811 (29.4)	1902 (29.3)	1909 (29.4)	
	24 to 35 m	6869 (26.8)	3815 (24.7)	3054 (29.9)		4102 (31.6)	2050 (31.6)	2052 (31.6)	
	36 to 48 m	7317 (28.5)	5788 (37.4)	1529 (15.0)		2865 (22.1)	1437 (22.2)	1428 (22.0)	
Sex (%)	Male	13,304 (51.8)	8079 (52.2)	5225 (51.2)	0.127	6706 (51.7)	3353 (51.7)	3353 (51.7)	1
	Female	12,364 (48.2)	7392 (47.8)	4972 (48.8)		6264 (48.3)	3132 (48.3)	3132 (48.3)	
Address (%)	Other	51 (0.2)	43 (0.3)	8 (0.1)	<0.001*	0 (0.0)	0 (0.0)	0 (0.0)	1
	Chedun	491 (1.9)	311 (2.0)	180 (1.8)		138 (1.1)	69 (1.1)	69 (1.1)	
	Dongjing	808 (3.1)	437 (2.8)	371 (3.6)		334 (2.6)	167 (2.6)	167 (2.6)	
	Fangsong	6453 (25.1)	4674 (30.2)	1779 (17.4)		3248 (25.0)	1624 (25.0)	1624 (25.0)	
	Guangfulin	20 (0.1)	17 (0.1)	3 (0.0)		2 (0.0)	1 (0.0)	1 (0.0)	
	Jiuting	3627 (14.1)	1920 (12.4)	1707 (16.7)		2176 (16.8)	1088 (16.8)	1088 (16.8)	
	Liiugang	238 (0.9)	175 (1.1)	63 (0.6)		36 (0.3)	18 (0.3)	18 (0.3)	
	Sheshan	1037 (4.0)	566 (3.7)	471 (4.6)		432 (3.3)	216 (3.3)	216 (3.3)	
	Shihudang	200 (0.8)	124 (0.8)	76 (0.7)		32 (0.2)	16 (0.2)	16 (0.2)	
	Sijing	3045 (11.9)	1625 (10.5)	1420 (13.9)		1818 (14.0)	909 (14.0)	909 (14.0)	
	Xiaokunshan	384 (1.5)	273 (1.8)	111 (1.1)		78 (0.6)	39 (0.6)	39 (0.6)	
	Xinbang	181 (0.7)	152 (1.0)	29 (0.3)		12 (0.1)	6 (0.1)	6 (0.1)	
	Xinqiao	2034 (7.9)	991 (6.4)	1043 (10.2)		1082 (8.3)	541 (8.3)	541 (8.3)	
	Yexie	478 (1.9)	328 (2.1)	150 (1.5)		116 (0.9)	58 (0.9)	58 (0.9)	
	Yongfeng	2436 (9.5)	1546 (10.0)	890 (8.7)		1294 (10.0)	647 (10.0)	647 (10.0)	
	Yueyang	2101 (8.2)	1217 (7.9)	884 (8.7)		1094 (8.4)	547 (8.4)	547 (8.4)	
	Zhongshan	2084 (8.1)	1072 (6.9)	1012 (9.9)		1078 (8.3)	539 (8.3)	539 (8.3)	

* p < 0·01.

**Fig. 2:**
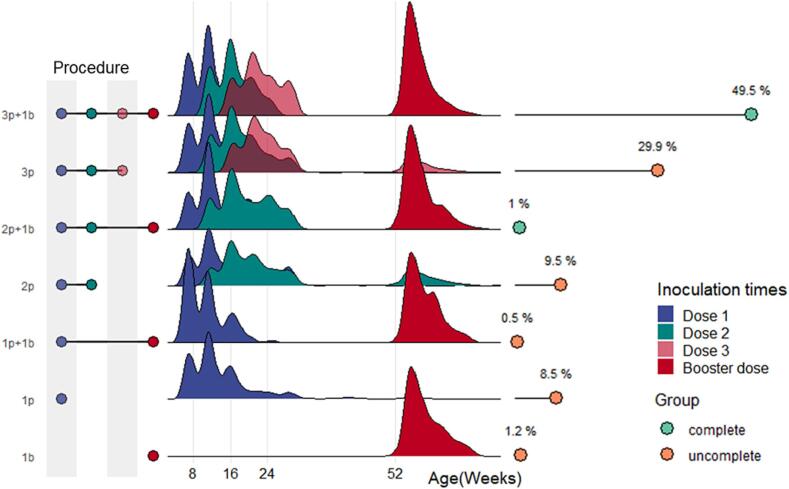
Distribution of PCV13 vaccination time for different doses. The distribution of vaccination time among children with different vaccination schedules and proportion of children vaccinated with different schedules are shown. The vaccination time of children is basically in line with the recommended vaccination program. 49.5% of children completed the full 3 + 1 doses before December 31, 2020.

Most of the children in Shanghai received PCV13 in accordance with the 3 + 1 vaccination program (Fig. 2), but some received only three doses of the initial formulation and did not receive a booster injection. According to the vaccination schedule, children who received 3p + 1b, 2p + 1b, or 3p were sorted to the complete vaccination group. Among the 10,197 children who were vaccinated with at least 1 dose of PCV13, 5,668 received 3p + 1b, 2,774 received 3p, and 94 received 2p + 1b. In total, 85.7% of the vaccinated children aged had received three doses of PCV13.

### 3.2 Vaccination and incidence rate

**Fig. 3:**
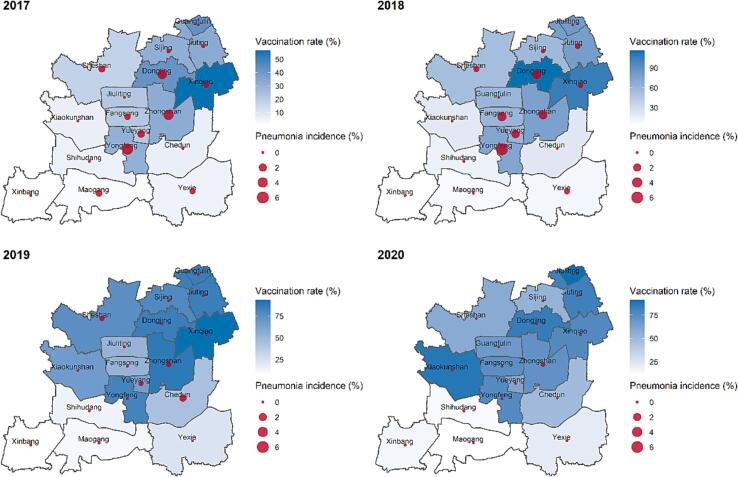
Variation tendency of PCV13 vaccination rate and all-cause pneumonia incidence from 2017 to 2020 in Shanghai’s Songjiang District**.** Vaccination rate and incidence of community-acquired pneumonia in each street town of Songjiang District, Shanghai from 2017 to 2020. The shade of blue represents the vaccination rate, and the size of the red dot represents the incidence of community-acquired pneumonia.

Since PCV13 was first approved by the Chinese government and became available on the Chinese market in 2017, the coverage of this vaccine has increased annually. In light of the SARS-Cov-2 epidemics, however, the number of children vaccinated with PCV13 has recently shown a slight decrease (Table 1). As shown in Fig. 3, the overall pneumonia incidence for the streets and communities of Shanghai’s Songjiang District decreased rapidly beginning in 2017. The vaccination rate in north Songjiang District was significantly higher than the south over the past few years, with vaccination rate of some streets and communities exceeding 90% in 2018 and 2019. Compared with that of 2017, when PCV13 was introduced, the overall vaccination rates of most streets and communities in the district increased by 20–30% across the study period. Meanwhile, pneumonia incidence showed an opposite trend, obviously trending downward in most regions of Songjiang District from 2017 to 2019. In the communities of this district, the incidence of pneumonia rapidly declined from 10 to 30% to 2.5–7.5% between 2017 and 2019. The top incidence rate was registered in the middle of 2017. Interestingly, we found that some streets and communities in south Songjiang District had particularly low risks of pneumonia.

### 3.3 Effectiveness of PCV13 vaccination

In unadjusted analyses performed prior to propensity-score matching, any course of PCV13 vaccination was associated with significantly lower risks, compared to no vaccine exposure, in the analyses of all-cause pneumonia (risk ratio, 0.985; 95% CI: 0.947 to 0.996), with homogeneous effects seen among outpatient children (risk ratio, 0.991; 95% CI: 0.986 to 0.997) and hospitalized children (risk ratio, 0.996; 95% CI: 0.992 to 0.999). Similarly, full course of PCV13 vaccination was associated with a significantly lower risk of all-cause pneumonia in children under 5 years old (risk ratio vs. no vaccine exposure, 0.990; 95% CI: 0.984 to 0.996), as well as in outpatient children (risk ratio, 0.990; 95% CI: 0.985 to 0.996) and hospitalized (risk ratio, 0.990; 95% CI: 0.984 to 0.996).

Analysis of the matched cohorts showed that during a mean follow-up of 1.76 years (interquartile range, 0.95–2.59), 581 all-cause pneumonias were documented (0.025 visits per person-years); of them, 398 visits were outpatient only, 17 were hospitalized only, and 166 had both outpatient and inpatient records (including 62 patients hospitalized after the same outpatient visit and 104 patients with separate outpatient and hospital encounters). When we controlled for gender and age, we obtained an HR of 82%, which means that the probability of a PCV13-vaccinated child being diagnosed with pneumonia in an outpatient clinic or hospital was 82% that of an unvaccinated child. The risk difference was −0.01064 (95% CI: −0.02208 to 0.00066), which means that PCV13 could prevent 1% of children from developing pneumonia. The NNH (number needed to treat) was 94 (95% CI: 45 to 990), which means that reducing one case of pneumonia requires that at least 94 children be vaccinated.

### 3.4 Stratified analysis

Considering that the number of doses may affect the results of the study, we further stratified the children of the vaccination group into those who had completed the entire vaccination process and those who had not. The calculated HR showed that the full (0.82, 95% CI: 0.69 to 0.98) and partial (0.84, 95% CI: 0.51 to 1.41) vaccination programs had relatively similar effects. Because relatively few vaccinated children did not complete the full program, the effect of partial vaccination programs was not statistically significant (Table 2).

**Table 2:** Subgroup analysis of PCV13 protection effectiveness.

Characteristics	Group	No Vaccine Exposure			Vaccine Exposure			Hazard Ratio (95% CI)
		Observation time (year)	No. of Events	Incidence density (1/1000 py)	Observation time (year)	No. of Events	Incidence density (1/1000 py)	
Vaccination procedure	Complete	10167.5	285	0.02803	10289.9	237	0.02303	0.82 (0.69–0.98) *
	Uncomplete	1164.6	32	0.02747	1163.7	27	0.02320	0.84 (0.51–1.41)
Hospitalization or not	Hospitalized	10647.2	97	0.00927	10526.5	68	0.00646	0.70 (0.51–0.95) *
	Outpatient	10177.5	280	0.02751	10301.9	229	0.02223	0.81 (0.68–0.96) *
Follow-up time	<1y	773.7	181	0.23393	754.8	135	0.17885	0.77 (0.62–0.97) *
	1 to < 2y	2729.1	81	0.02968	2706.4	76	0.02808	0.95 (0.7–1.3)
	2-3y	4555.7	19	0.00417	4595.2	22	0.00479	1.15 (0.62–2.12)

* p < 0·01.

**Fig. 4:**
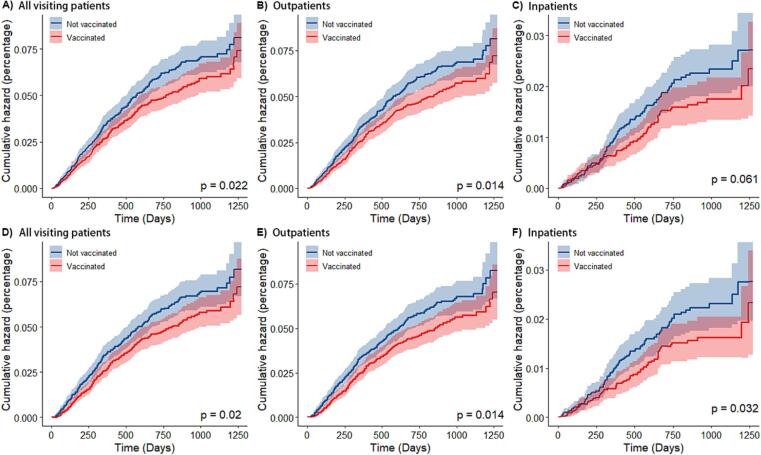
Cumulative hazard of two outcomes. Cumulative incidence curves (1 minus the Kaplan-Meier risk) for the various outcomes are shown, starting from the day of administration of the first dose of vaccine. Shaded areas represent 95% confidence intervals. In figure A), B) and C), the children in the vaccinated group included children who received at least one dose of PCV13, while in figure D), E) and F), the children in the vaccinated group only included children who received at least 3 doses of PCV13.

PCV13 was associated with significantly lower risks, compared to no vaccine exposure, in the analyses of all-cause pneumonia, outpatient pneumonia, and hospitalized pneumonia (Fig. 4). The estimated vaccine effectiveness was 19% (95% CI: 3 to 32) for all-cause pneumonia visits, 20% (95% CI: 5 to 33) for outpatient visits, and 29% (95% CI: 3 to 48) for hospitalization, in children vaccinated with at least one dose of PCV13. The protective effectiveness of PCV13 was found to be higher for hospitalized pneumonia (30%, 95% CI: 5% to 49%) than for outpatient pneumonia (19%, 95% CI: 4% to 32%).

Our results showed that as the observation time increased, the protection rate decreased slightly: It was highest at 1 year after vaccination and no protective effect was observed after 2 years. We suspected that this may reflect that the incidence of pneumonia is high in children is 0–2 years old and thereafter gradually decreases with age (Table 2 and Fig. 4).

## 4 Discussion

This study is the first cohort study linking real-world electronic immunization records and health outcome databases to evaluate the effectiveness of PCV13 for preventing hospitalization, outpatient, and all-cause pneumonia among children living in Songjiang District of Shanghai, China. As PCV13 vaccination is currently self-paid in China, the overall coverage rate of PCV13 in China is low and shows obvious regional differences. Shanghai has a high per capita income, so the vaccination rate has risen rapidly (from 13.87% in 2017 to 61.43% in 2020). However, there are coverage differences between streets (Fig. 3)[32]. In the present study, children who had completed 3p + 1b, 2p + 1b, or 3p vaccination doses were regarded as the complete vaccination group, because a previous study showed that PCV13 administered in a 3p + 1b versus 2p + 1b dose schedule was immunogenic and well tolerated in healthy Chinese infants, and likely to protect against PCV13 serotypes[33]. Our result shows that the immunization procedures for children in Shanghai are standardized and a high proportion of children complete the entire vaccination process.

After we controlled for baseline characteristics and residential communities of the two groups, we found that PCV13 vaccination could reduce the outpatient visit rate and hospitalization rate of CAP in children under 5 years of age in China. *Streptococcus pneumoniae* infection of the lungs can lead to pneumococcal pneumonia, and invasion of sterile parts of the body can lead to more severe invasive pneumococcal diseases (IPDs). Previous studies showed that PCV13 had a good effect in preventing serotype IPD.A matched case-control study in the United States [34] showed that the protection rate of PCV13 against IPD was 60.2% (95% CI: 46.8% to 70.3%). A study in the United Kingdom [35] showed that the protection rate of PCV13 against PCV7 serotype IPD was 92.0% (95% CI: 81.7% to 96.7%), and the protection rate against the other six serotypes was 73.7% (95% CI: 31.1% to 89.9%). The relatively low effectiveness for preventing all-cause pneumonia, compared to IPD, reflects that all-cause pneumonia includes those caused by viruses, mycoplasma, and other pathogens. According to the literature, bacterial pneumonia in China accounts for only about 30.9% of all-cause pneumonia [36]. Thus, it is important that PCV13 can prevent only the portion of bacterial pneumonia caused by 13 serotypes of *Streptococcus pneumoniae*. Our results showed that PCV13 has a better effect on hospitalized pneumonia compared to outpatient pneumonia. This may reflect that inpatients are more severely ill, and the proportion caused by bacterial infection may be higher than that among outpatients.

Some longitudinal studies that have shown that PCV13 can reduce the global incidence of CAP in children. In the United States, the CAP hospitalization rate dropped significantly after the introduction of PCV13, by 21% and 17% among those younger than 2 years and 2–4 years old, respectively [37]. A French study of children aged 1 month to 15 years showed that the number of CAP cases was 16% lower after the application of PCV13, with infant cases decreasing by 32% [38]. A prospective multicenter study also performed in France showed that a sustained reduction in CAP cases was observed 5 years after PCV13 implementation: The number of cases of overall CAP decreased by 25.4% and those of hospitalized CAP decreased by 30.5% [39]. An observational hospital-based surveillance study in Japan showed that the CAP hospitalization rates per 1000 child-years were 17.7, 14.3, and 9.7 in children aged < 5 years in 2008, 2012, and 2018, respectively, and the incidence of hospitalized CAP in children was significantly reduced after the introduction of PCV13 in Japan [40].

In the present study, we found that PCV13 vaccination can significantly reduce the incidence of clinical all-cause pneumonia in children under 5 years of age, whether outpatient or inpatient. This conclusion is consistent with other cohort evidence. An observational study conducted by Zhang et al. in Suzhou, China found a vaccine effectiveness (VE) of 61% for a first hospital visit with community acquired pneumonia associated with PCV13 serotype carriage (VT-CAP) and 18% for clinically defined CAP in Suzhou, China [41]. Although the coverage rate in newborns has risen quickly in Songjiang District, it has been only 3 years since the introduction of vaccination. Moreover, the effect of herd protection is difficult to observe when coverage in the whole population is low. [42], [43] We speculate that if the coverage rate remains at the current level or continues to rise, we will likely observe a more pronounced decline in the incidence of pneumonia and even herd-protection effects in the future.

The limitations of our study include the following: Its scope was limited to Songjiang District, Shanghai, and therefore the extrapolation of our results is open to question. Affected by integrality of all-cause pneumonia medical records, a few numbers of children infected by pneumonia may fail to detect in our study, underestimating the incidence of pneumonia. The outcome was defined as clinical pneumonia, the judgment of which depends on the diagnosis of a clinician. Some of the baseline information was controlled for, but the possibility of other confounding factors cannot be ruled out. Since fewer children received vaccination procedures other than 3p + 1b, we were unable to identify differences in the protective effects of the different vaccination procedures.

## 5 Conclusions

In conclusion, this study estimates the effectiveness of the PCV13 vaccine for preventing all-cause community acquired pneumonia in a real-world setting, and suggests that this effectiveness is high for serious outcomes (e.g., inpatient). Our results provide strong support for expanding the availability of PCVs to children in China, and may offer a basis for evaluating the economic impact of including PCV13 in the national immunization program.

## 6 Declarations

### 6.1 Ethics approval and consent to participate

This study was reviewed and approved by Medical Research Ethics Committee, School of Public Health, Fudan University (International registration number: IRB00002408 & FWA00002399), approval number is IRB# 2019-11-0789. Consent to participate was not applicable.

### 6.2 Author’s contributions

JT and WW designed the study. YG and CX collected the data. JT and LY extracted the data and constructed the database. JT and BZ analyzed the data and drafted the manuscript. WW and LY conducted critical revisions of the manuscript. All authors read and approved the final manuscript.

## Declaration of Competing Interest

The authors declare that they have no known competing financial interests or personal relationships that could have appeared to influence the work reported in this paper.

## Data Availability

Data will be made available on request.
